# The Kinetics of Force-Induced Cell Reorganization Depend on Microtubules and Actin

**DOI:** 10.1002/cm.20439

**Published:** 2010-02-26

**Authors:** Alexandra M Goldyn, Peter Kaiser, Joachim P Spatz, Christoph Ballestrem, Ralf Kemkemer, Pekka Lappalainen

**Affiliations:** 1 Department of New Materials and Biosystems, Max Planck Institute for Metals ResearchHeisenbergstr. 3, Stuttgart, Germany; 2 Department of Biophysical Chemistry, University of HeidelbergIm Neunheimer Feld 253, Heidelberg, Germany; 3 Wellcome Trust Centre for Cell-Matrix Research, Faculty of Life Sciences, University of ManchesterManchester, England, United Kingdom

**Keywords:** forces, migration, actin, microtubules, myosin

## Abstract

The cytoskeleton is an important factor in the functional and structural adaption of cells to mechanical forces. In this study we investigated the impact of microtubules and the acto-myosin machinery on the kinetics of force-induced reorientation of NIH3T3 fibroblasts. These cells were subjected to uniaxial stretching forces that are known to induce cellular reorientation perpendicular to the stretch direction. We found that disruption of filamentous actin using cytochalasin D and latrunculin B as well as an induction of a massive unpolarized actin polymerization by jasplakinolide, inhibited the stretch-induced reorientation. Similarly, blocking of myosin II activity abolished the stretch-induced reorientation of cells but, interestingly, increased their motility under stretching conditions in comparison to myosin-inhibited nonstretched cells. Investigating the contribution of microtubules to the cellular reorientation, we found that, although not playing a significant role in reorientation itself, microtubule stability had a significant impact on the kinetics of this event. Overall, we conclude that acto-myosin, together with microtubules, regulate the kinetics of force-induced cell reorientation. © 2010 Wiley-Liss, Inc.

## Introduction

Mechanical forces are critical for normal development and maintenance of many tissues [Alenghat and Ingber, 2002; Janmey and McCulloch, 2007] and are of importance in various pathological processes such as atherosclerosis, osteoporosis, and cancer [Ingber, 2003; Wang and Thampatty, 2006; Suresh, 2007]. Cells, as the basic unit of tissues, are able to sense mechanical stresses and adapt their functions and morphologies accordingly. The cell cytoskeleton, as a dynamic network of filamentous actin (f-actin), microtubules (MTs), and intermediate filaments, is known to be a key element for force-induced cellular responses [Goode et al., 2000; Geiger et al., 2001]. To satisfy their complex functions in mechano-responses the cytoskeletal elements must be collectively regulated [Goode et al., 2000; Etienne-Manneville, 2004]. The acto-myosin system is particularly well studied and it is widely accepted that it plays a crucial role in converting external forces into biological responses [Alenghat and Ingber, 2002; Cai and Sheetz, 2009]. For example, cells exposed to cyclic stretching reorient their cell body with their actin cytoskeleton perpendicular to the direction of stretch [Dartsch and Betz, 1989; Wang et al., 2001; Jungbauer et al., 2008; Goldyn et al., 2009]. However, F-actin disruption prevents the stretch-induced reorientation [Goldyn et al., 2009].

The F-actin network is one of several components relevant to force-sensing and force-transduction in cells. It is known that MTs can also influence the force machinery and their dynamics are essential for coordinated cell migration [Wehrle-Haller and Imhof, 2003; Etienne-Manneville, 2004].

In addition to actin, MTs have been demonstrated to be mechano-sensitive. For example, MT polymerization was stimulated by a single pulling or stretching of cells [Suter et al., 1998; Kaverina et al., 2002] and the disruption or hyperpolymerization of MTs inhibited shear flow-induced morphological changes [Malek and Izumo, 1996; Hu et al., 2002]. For cyclic stretch experiments, we recently demonstrated that intact MTs were not necessary for cellular reorientation [Goldyn et al., 2009]; however, it is still unclear whether MTs can control the kinetics of cellular reorientation under force.

In order to study the contribution of acto-myosin and MTs in the force-induced reorientation of cells, we performed uniaxial cell stretching experiments using NIH3T3 fibroblasts. To reveal the particular influence of the two cytoskeletal components (actin and MTs), we exposed the cells to cytoskeleton-disturbing drugs. We then analyzed cell migration, the kinetics of cell orientation, and the degree of actin and MT reorganization. Our data show that MTs had a significant effect on the kinetics of the cellular and acto-myosin network reorganization. Moreover, reduced migration of myosin II-inhibited cells was restored to nontreated migration levels by application of cyclic stretching.

## Materials and Methods

### Cell Culture and Pharmacological Cytoskeleton Inhibitors

NIH3T3 mouse fibroblasts (from DSMZ, Braunschweig, Germany) were cultured in Dulbecco's modified eagle medium (DMEM) (Invitrogen, Karlsruhe, Germany) supplemented with 10% fetal calf serum (FCS; Invitrogen). Cells for experiments were used with a passage number lower than 25.

Concentrations of 3 μM taxol, 3 μM nocodazole, 1 μM cytochalasin D, 1.5 μM latrunculin B, 10 μM blebbistatin (all from Sigma-Aldrich, Munich, Germany), and 25 nM jasplakinolide (Calbiochem, Merck, Darmstadt, Germany), were used and cells were preincubated for about 30 min at standard cell culture conditions before the experiments were started. For an overview of pharmacological cytoskeleton inhibitors refer to Table SI, Supporting Information.

### Cell Fixation and Immunofluorescence Staining

Cells were fixed with 3.7% paraformaldehyde (PFA) (Serva, Heidelberg, Germany) supplemented with 0.05% glutaraldehyde (Merck, Darmstadt, Germany) for 10 min. For visualization of microtubules (MTs), mouse monoclonal anti-β-tubulin (clone TUB2.1) (Sigma), and goat anti-mouse Alexa Fluor 350 (Invitrogen) were used in a 1:300 or 1:400 dilution, respectively. For actin staining, Alex Fluor 488 phalloidin (Invitrogen) was used in a 1:60 dilution. Cells were analyzed using an upright light microscope (AxioImager Z1, W-Plan Apochromat 63x/1.0 VIS-IR water immersion objective) (Zeiss, Jena, Germany) equipped with an AxioCam MRm CCD camera (Zeiss). The fluorescent images of fixed cells were contrast enhanced.

### Stretching Experiments

Stretching experiments were performed in complete medium supplemented with 1% penicillin-streptomycin (Invitrogen) at standard cell culture conditions (37°C, 5% CO_2_) as described in detail by Jungbauer et al. [ 2008]. Prior to each experiment, 50 cells/mm^2^ were plated on elastic poly(dimethylethylensiloxane) (PDMS) membranes, which were coated with 20 μg/ml fibronectin. The cells were then let to adhere over night. Cyclic stretching was performed at a frequency of 1 Hz and 8 % of linear stretch amplitude. The stretching device was mounted on an inverted light microscope (AxioVert 200M, 10x/0.25Ph1 objective or 20x/0.25Ph1 objective, Zeiss, Jena, Germany), equipped with a CCD-camera (PCO Sensicam QE, Kelheim, Germany). A self-developed software routine embedded in Image Pro 6.2 (Media Cybernetics, Bethesda, USA) was used to control the instrument during time-lapse phase contrast imaging.

### Analysis of Cell Orientation, Size, and Elongation

#### Cell orientation

**Fig. 1:**
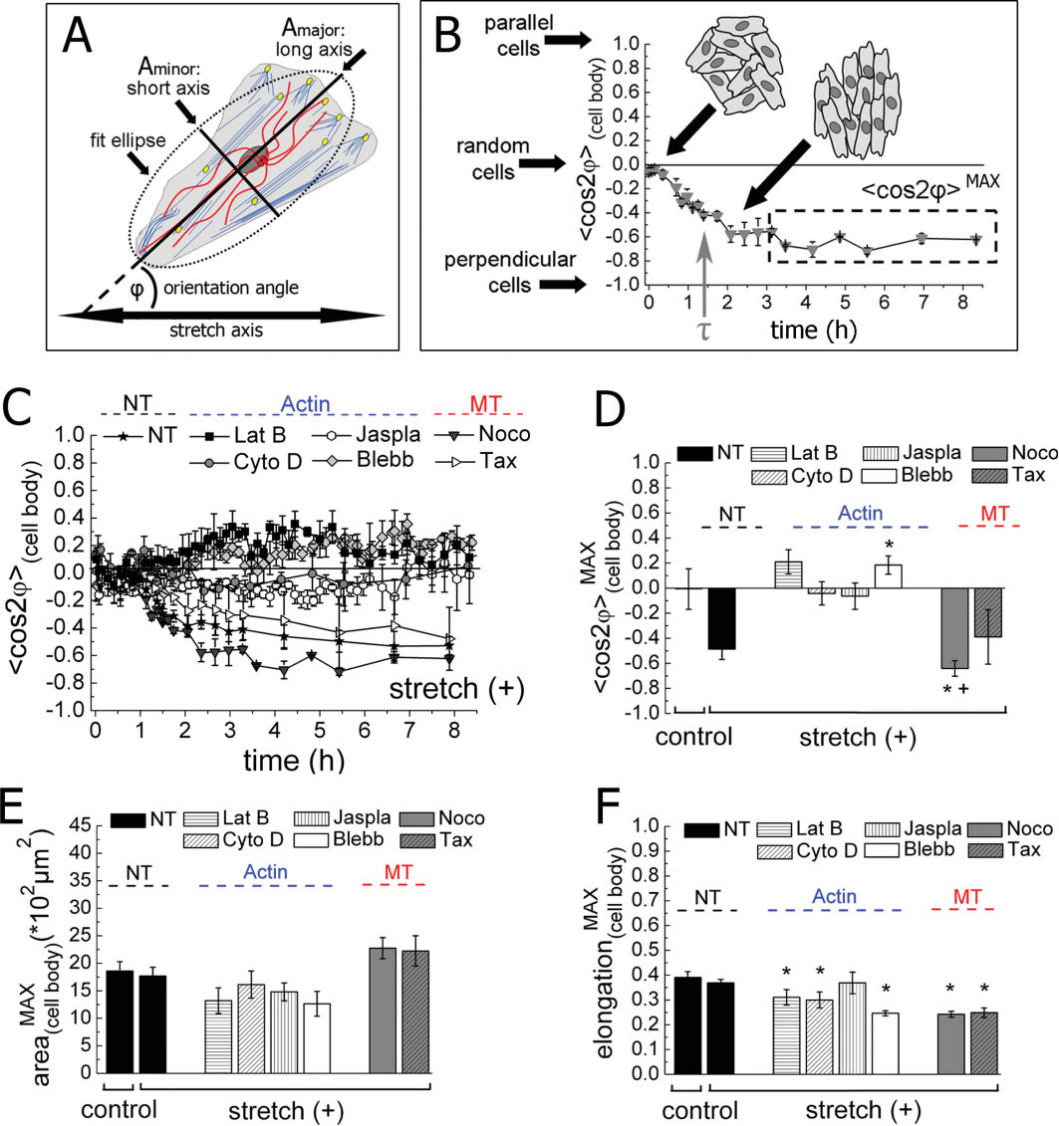
Influence of pharmacological cytoskeleton inhibitors and uniaxial cyclic stretch on cellular morphology. **A**: Schematic representation of cell analysis. Cell orientation was calculated by fitting an ellipse to a cell outline and measuring the orientation angle, φ, between the long axis of the cell and the stretch direction. Cell elongation was calculated from the long and short axes of the ellipse. Cell area was given directly by the outlined cell. **B**: Example for a time course of the reorientation response of NIH3T3 cells upon uniaxial cyclic stretching of 8% at 1 Hz. The mean value of 〈cos 2φ〉 = 1 indicates that the cells are orientated parallel; the minimum of −1 indicates a perpendicular alignment with respect to the stretch direction. A value of 〈cos 2φ〉 = 0 corresponds to a random orientation of cells. The mean value for the cell reorientation from *t* = 3 to 8 h (dotted box in the graph) was calculated to obtain a steady state, maximum value (〈cos 2φ〉^MAX^_(cell_body)_). The characteristic time (τ) describes the time until 〈cos 2φ〉 reaches approximately 63% of the maximum reorientation and is indicated in the figure by the gray arrow. **C**: Time course of the mean reorientation of cells upon uniaxial cyclic stretching of 8% at 1 Hz (stretch (+)) under the indicated conditions. **D**: Quantification of the maximum mean cell reorientation (〈cos 2φ〉^MAX^_(cell_body)_) under the conditions indicated. Control indicates nonstretched conditions and stretch (+) the application of cyclic stretch. The disruption of microtubules (MTs) with nocodazole enhanced the maximum cellular reorientation in comparison with nontreated stretched cells (*, *P* < 0.05) and compared to cells with stabilized microtubules (+, *P* < 0.05). Blebbistatin-treated cells oriented significantly more parallel compared to nontreated, nonstretched (control) cells (*, *P* < 0.05). **E**: Quantification of the maximum mean cell area (area ^MAX^_(cell_body)_) under the conditions indicated. The cell area for cells with a disturbed acto-myosin or MT network did not significantly vary compared to nontreated stretched conditions. **F**: Quantification of the maximum mean cell elongation (elongation(^MAX^_(cell_body)_) under the conditions indicated. A value of 0 corresponds to a spherical cell area; a value close to 1 represents an extremely elongated cell. The cell elongation was in most cases significantly decreased compared to nontreated stretched conditions (*, *P* < 0.05). NT, nontreated cells; Actin, cells with a disturbed actin network (Lat B, latrunculin B; Cyto D, cytochalasin D; Jaspla, jasplakinolide; Blebb, blebbistatin); MT, cells with a modified microtubule network (Noco, nocodazole; Tax, taxol).

Cell orientation was measured as described previously [Jungbauer et al., 2008]. Briefly, an ellipse was fitted to each cell outline. The orientation angle, φ, of the long axis of the ellipse with respect to the stretch direction was measured (Fig. 1A). For control experiments (nonstretched condition) the *x*-axis of the images was chosen as reference direction. The orientation angle φ is transformed into the orientation parameter cos 2φ. A value of 〈cos 2φ〉 = 0 corresponds to cells that are in average randomly oriented, 〈cos 2φ〉 = 1 if cells are in average parallel oriented, and 〈cos 2φ〉 = −1 if they are perpendicularly oriented with respect to the stretch axis (Fig. 1B).

#### Cell elongation

The elongation was calculated from the major axis (*A*_maj_) and the minor axis (*A*_min_) of the ellipse fitted to the cell outline (Fig. 1A):


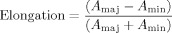


The cell elongation is thus mapped to a scale between 0 (spherical) and 1 (infinitely thin line).

#### Cell area

To obtain the cell area, the cell shape/morphology was manually outlined and is displayed in mm^2^. Mean values of an ensemble of cells per indicated time points are presented.

For the evaluation of the maximum value of orientation, area, and elongation, the mean value from the last 5 h of the data set was calculated (Fig. 1B).

For each experimental condition, about 50–70 cells from three to four independent experiments were analyzed.

### Characteristic Time τ of Cellular Reorientation

The characteristic time τ was derived from a least-square fit of the experimental data to the following equation (for details of the analysis see [Kemkemer et al., 1999]:


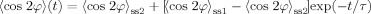


The steady state orientation 〈cos 2φ〉_ss1_ is the initial orientation at time point zero (random orientation under nonstretched conditions) changing to a new steady orientation state 〈cos 2φ〉_ss2_ at a later time point (perpendicular orientation of the cells with respect to the stretch direction).

The characteristic time τ indicates the time of stretch-induced realignment and describes how fast the cells reorient upon cyclic stretch application. More precisely, τ gives the time until the orientation parameter 〈cos 2φ〉 reached 1/*e* (∼63%) of the maximum (final) orientation.

### Analysis of Actin Stress Fibers and Microtubules

**Fig. 2:**
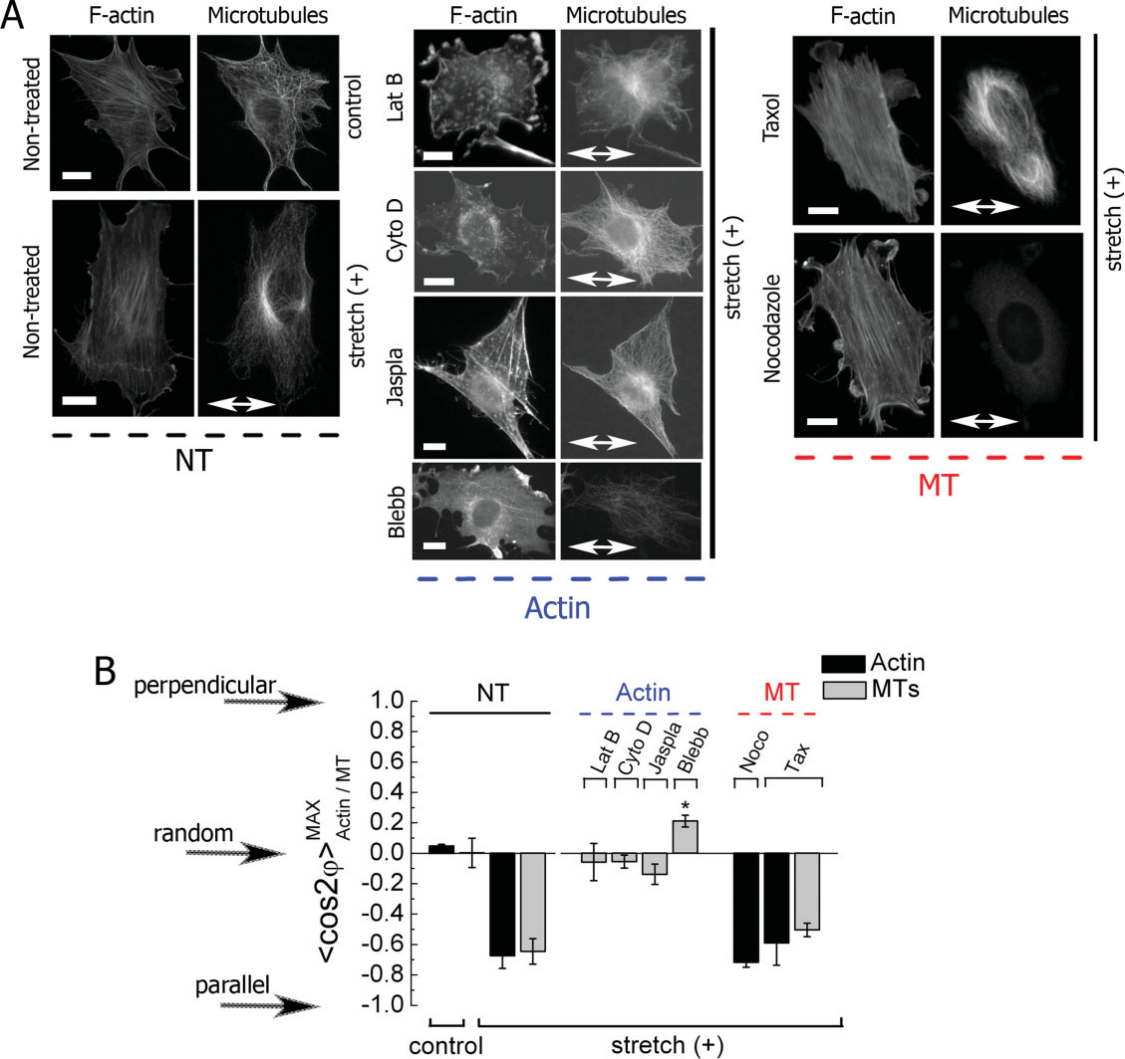
The actin and the microtubule network align perpendicular to the stretch direction. **A**: Actin and microtubule (MT) staining after 3 h of cyclic stretching. Actin was stained using phalloidin and MTs were visualized by an anti-β-tubulin antibody. Control indicates nonstretched conditions and stretch (+) the application of cyclic stretching. Actin stress fibers and MTs oriented perpendicular to the stretch direction (white double-headed arrow) under nontreated conditions and despite nocodazole or taxol treatment. MT reorientation was dependent on the orientation of the actin cytoskeleton and did not occur in actin-treated cell (Scale bars: 10 μm). **B**: Quantification of f-actin and MT orientation under indicated conditions after 3 h of cyclic stretch (1 Hz, 8 % amplitude). Cellular structures were analyzed by using Fast Fourier Transformation (FFT) analysis of cell subareas to yield 〈cos 2φ〉 values. A value of -1 means a perpendicular orientation of the investigated structures, a value of 1 indicated an average parallel orientation and 0 an average random orientation. The alignment of f-actin and MTs was significantly higher under nontreated, nocodazole-treated, and taxol-treated stretching conditions compared to nontreated, nonstretched conditions (control). Cells treated with actin-interfering substances showed no differences in stretch-induced alignment of MTs compared to the nontreated, nonstretched control. A significant parallel MT orientation was observed for cells treated with blebbistatin (*,*P* < 0.05). NT, nontreated cells; Actin, cells with a disturbed actin network (Lat B, latrunculin B; Cyto D, cytochalasin D; Jaspla, jasplakinolide; Blebb, blebbistatin); MT, cells with a modified microtubule network (Noco, nocodazole; Tax, taxol). [Color figure can be viewed in the online issue, which is available at www.interscience.wiley.com.]

For analysis of actin stress fibers and MT orientation, a self-developed macro embedded in ImageJ (http://rsb.info.nih.gov/ij/) was used. Subareas of a cell were analyzed by texture analysis via a Fast Fourier Transformation (FFT) in analogy to Kemkemer et al. [Kemkemer et al., 2000]. In brief, each cell picture was divided into many small squares (64 × 64 pixels). For each square, a FFT was performed and the resulting image was further analyzed to measure the mean orientation of fibers within the square. The measured angle of orientation (φ) of actin stress fibers or MTs within the analyzed square was plotted into the image where the *x*-axis of the image indicates the direction of stretch or the arbritary *x*-axis in the nonstretched case. The orientation angle φ is transformed into the orientation parameter cos 2φ. The mean value (〈cos 2φ〉) of all analyzed cell subareas corresponds to the mean orientation parameter for actin stress fibers or MTs (Fig. 2B). For each experimental condition, at least 30 cells from three independent experiments were evaluated.

### Correlation Analysis

**Fig. 3:**
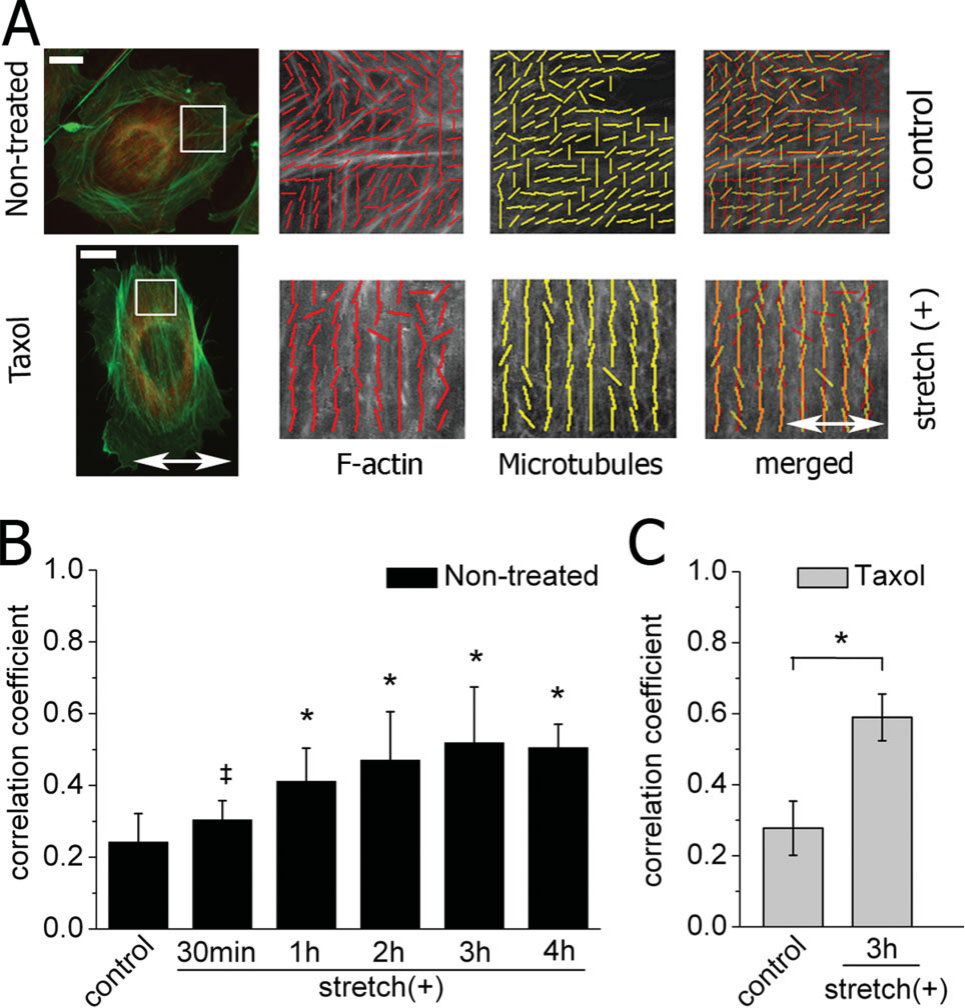
Actin stress fibers and microtubules are coaligned in cells subjected to cyclic stretching. **A**: Analysis of actin stress fiber and microtubule (MT) orientation. The mean orientation of fibers was determined for each cellular subarea (white square). The orientation lines for actin stress fiber (= red) or MT (= yellow) alignment were plotted into the images with respect to the *x*-axis. The line overlays for actin stress fibers and MTs were merged into a new image for visualization of coorientation. The top row shows an example of the analysis of a nontreated, nonstretched (control) cell. The bottom row demonstrates the analysis of a taxol-treated stretched (+) cell. The white double-headed arrow indicates the direction of stretch; scale bars: 10 μm. **B, C**: Quantification of f-actin and MT coalignment under nontreated (B) and taxol-treated (C) conditions at indicated time points after cyclic stretching (1 Hz, 8 % amplitude). A correlation coefficient between f-actin and MTs orientation was calculated from the angles of the orientation lines with respect to the *x*-axis (= stretch direction in the case of cyclic stretch application). A value of 1 for the correlation coefficient would mean coalignment of f-actin and MTs, a value close to 0 would mean random, independent organization of f-actin and MTs. Cyclic stretching leads to a two-fold increase in the correlation coefficient under nontreated and taxol-treated conditions compared to nonstretched (control) conditions (*, *P* < 0.05, *t*-test after Fisher Transformation).

The orientation of actin stress fibers and MTs was determined as described above. For each cell, lines indicating the measured local mean orientation of actin stress fibers or MTs were plotted within the analyzed cell subarea for visualization (red = actin, yellow = MTs) (Fig. 3A). Both orientation lines were merged into a new image to indicate coorientation (merged = orange). For each cell, the pair-wise correlation coefficient for the local orientation angles of the actin stress fibers and MTs with respect to the stretch direction was calculated by OriginPro 8G software (OriginLab Corporation, Northampton, USA). A value of 1 for the correlation coefficient would mean a perfect coalignment of the investigated elements within the analyzed area, a value close to 0 would mean no correlation between the organization of actin stress fibers and MTs. For each experimental condition, a minimum of five cells were evaluated and for each cell at least four subareas were analyzed.

### Analysis of Cell Migration

Cell migration was analyzed by tracking the cell nucleus using the Manual Tracking Plug-In for ImageJ. The tracking patterns and the accumulated distance (μm/8 h) of migration were recorded. The orientation of cell migration was determined as the ratio of root mean square displacements in *y*- and *x*-direction, where the *x*-direction is the direction of applied stretch or the arbitrary the *x*-axis in the nonstretched case and *n* is the number of time-points that were analyzed:


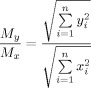


**Fig. 4:**
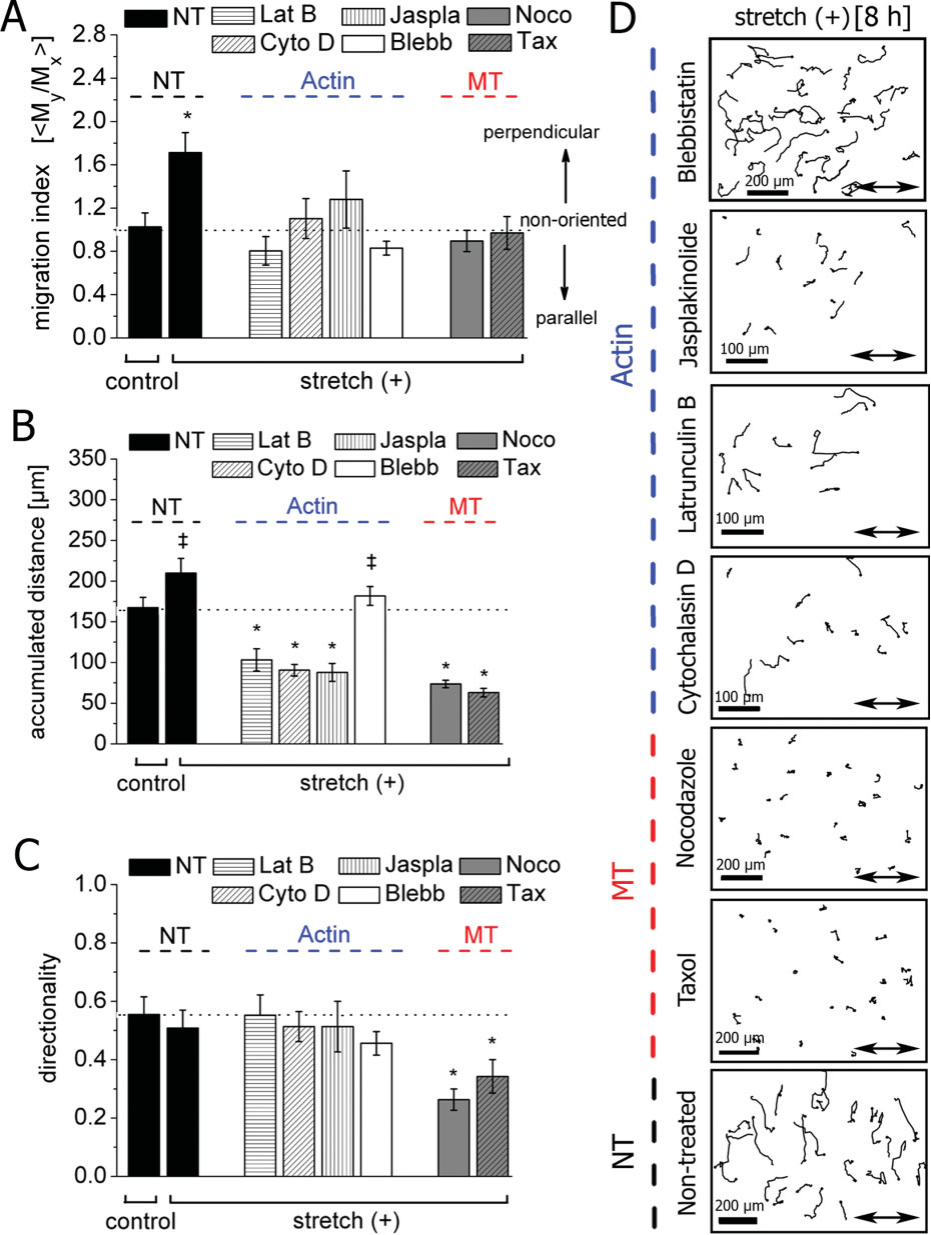
Stretch-induced oriented migration requires an intact actin and microtubule cytoskeleton. **A**: Quantification of oriented mean cell migration was performed over 8 h under the conditions indicated. A migration index (*M*_*y*_/*M*_*x*_) for the oriented migration was calculated. *M*_*y*_/*M*_*x*_ = 1 indicates no preferred migration direction (black dotted line in the graph), for *M*_*y*_/*M*_*x*_ < 1 cell migrates preferentially parallel to the stretch direction, and for *M*_*y*_/*M*_*x*_ > 1 cell migrates preferentially perpendicular to the stretch direction. Cell migration was observed to be perpendicular to the stretch axis only for nontreated (NT) stretched [stretch (+)] cells [〈*M*_*y*_/*M*_*x*_〉 ≍ 1.71, *,*P* < 0.05 compared to microtubule (MT)-treated and actin-treated (Actin) cells (〈*M*_*y*_/*M*_*x*_〉 ≍ 1]. **B**: Accumulated distance of cell migration. Cyclic stretching did not significantly change the overall accumulated distance of cell migration under nontreated conditions (‡, *P* > 0.05). Distance of cell migration was reduced for all drug-treated conditions compared to nontreated cells except for blebbistatin-treated cells (‡, *P* > 0.05). Blebbistatin-treated cells migrated with similar distances as nontreated cells (‡, *P* > 0.05). **C**: Cyclic stretching did not significantly change the directionality of migration (= euclidean distance/accumulated distance) for nontreated and cells with disrupted acto-myosin system compared to nontreated control cells. A value of 1 for the directionality would mean that cells migrate in a straight line. Directionality of cellular movement was dramatically reduced by stabilization (taxol treatment) or disruption (nocodazole treatment) of MTs (*, *P* < 0.001). **D**: Examples of cell migration tracks. Tracks of migrating cells were recorded for 8 h under the conditions indicated. Migration was abolished after treatment of cells with taxol or nocodazole, which, respectively stabilize or disrupt MTs. Cell migration was reduced for cells with disrupted acto-myosin system. Cell migration was observed to be perpendicular to the stretch axis only in nontreated stretched cells. The direction of stretch is indicated by the black double-headed arrow. NT, nontreated cells; Actin, cells with a disturbed actin network (Lat B, latrunculin B; Cyto D, cytochalasin D; Jaspla, jasplakinolide; Blebb, blebbistatin); MT, cells with a modified microtubule network (Noco, nocodazole; Tax, taxol). [Color figure can be viewed in the online issue, which is available at www.interscience.wiley.com.]

Thus, a migration index of *M*_*y*_/*M*_*x*_ = 1 means that the cell migrates with no preferred direction, for *M*_*y*_/*M*_*x*_ < 1 the cell migrates preferentially parallel to the stretch direction, and for *M*_*y*_/*M*_*x*_ > 1 the cell migrates preferentially perpendicular to the stretch direction (Fig. 4A). The directionality of migration was analyzed using the Chemotaxis and Migration Tool 1.01 for ImageJ. A value of 1 for the directionality would mean that a cell migrates in a straight line. For each experimental condition a minimum of 30 cells were analyzed from at least three independent experiments and mean values are given.

### Statistical Analysis

All data were expressed as means ± s.e.m. OriginPro 8G software was used for statistical analysis (*t* test, Fisher Transformation). Differences were considered statistically significant when the calculated *P* value was less than 0.05.

## Results

### Acto-Myosin Interfering Drugs Block Stretch-Induced Cellular reorientation; Drugs Affecting Microtubules Alter the Kinetics of Cell Reorientation

For the application of directional forces, we used a customized stretching device which allows uniaxial cyclic stretching of adherent cells when plated on flexible substrates [Jungbauer et al., 2008; Goldyn et al., 2009]. Nontreated cells reorient perpendicular to the direction of cyclic strain [Jungbauer et al., 2008; Goldyn et al., 2009]. For each experiment, we analyzed cell body orientation, cell area, and cell elongation. The orientation parameter (cos 2φ) was calculated for each single cell (Fig. 1A) and resulted in information about the mean orientation (〈cos 2φ〉) of a cell ensemble (Fig. 1B). The actin cytoskeleton (indicated as “Actin” or “Actin-treated” in the according paragraphs and figures) was modified by adding latrunculin B, cytochalasin D, jasplakinolide, and blebbistatin to the cell culture media. The microtubule (MT) network (indicated as “MT” or “MT-treated” in the relevant paragraphs and figures) was influenced by using nocodazole and taxol.

We first tested the effect of drugs that affect the integrity of f-actin and MTs on the kinetics of cellular reorientation by determining the characteristic time τ required for complete reorientation (Figs. 1B and 1C). As an example for the dynamic cell reorganization, the orientation parameter (〈cos 2φ〉) is plotted over time in Figure 1C (a complete overview of the nonstretched control data sets is given in Figure S1, Supporting Information). Stretch-induced reorganization of cells treated by actin-disrupting drugs (latrunculin B, cytochalasin D) or by drugs that inhibit myosin II function (blebbistatin) was blocked; cells with depleted MTs or with stabilized MTs were still able to reorient perpendicular to the stretch direction (Figs. 1C and 1D). The characteristic time τ was on average about 100 min for nontreated stretched cells. The disruption of MTs led to a decreased τ of 70 min and thus increased the overall speed of reorientation (1.4 times faster than nontreated cells). In contrast, stabilization of MTs using taxol caused an increase in τ to 290 min; these cells turned four times more slowly than nontreated cells (Fig. 1C and Table I).

**I:** Characteristic Time τ for the Stretch-Induced Cellular Reorientation is Increased for Cells with Disrupted Microtubules

Treatment	Characteristic time τ (min)	Characteristic time τ (h)	Ratio of (characteristic) reorientation time (normalized to nocodazole)
Nocodazole	70 ± 7	≍1.2	1
Non-treated	100 ± 9	≍1.7	1.4
Taxol	290 ± 100	≍4.8	4

The characteristic time τ describes the time of the cell reorientation process. It indicates the time until the mean orientation parameter (〈cos2φ〉) reaches the value of 1/e (approximately 63%) of the maximum mean orientation (for details see the Materials and Methods section). Note that cells without microtubules (nocodazole-treated) reorient faster than non-treated cells or cells with stabilized microtubules (taxol-treated).

We next measured the maximum orientation cells can reach (〈cos 2φ〉^MAX^) by averaging the values of the last 5 h of stretch application (Fig. 1B). Stretching under nontreated conditions led to the reorientation of initially randomly oriented cells (〈cos 2φ〉 ≍ 0) to an alignment of cells perpendicular to the stretch axis (〈cos 2φ〉^MAX^ ≍ −0.5). None of the cells treated with cytochalasin D, latrunculin B (actin disrupting), actin stabilizing (jasplakinolide), or blebbistatin (myosin II inhibition) were able to reorient perpendicular upon stretching. As seen previously [Goldyn et al., 2009], all of the cells that were treated with MT-disrupting drugs (nocodazole) or MT stabilizing drugs (taxol) readily repolarized (Figs. 1C and 1D). However, their maximum degrees of reorientation differ significantly (〈cos 2φ〉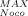 ≍ −0.64 and 〈cos 2φ〉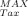 ≍ −0.39, +, *P* < 0.05). Remarkably, the degree of reorientation of cells depleted of MTs was also significantly higher than for nontreated cells (〈cos 2φ〉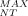 ≍ −0.5; *, *P* < 0.05; Fig. 1D).

Cells treated with substances that interfere with f-actin organization showed no stretch-induced cell reorientation (〈cos 2φ〉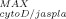 ≍ −0.05). Interestingly, cells with inhibited myosin II function, showed minor but significant cell alignment parallel to the stretch direction (〈cos 2φ〉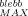 ≍ 0.2) (Fig. 1D; *, *P* < 0.05; Fig. S2A, Supporting Information).

We investigated further if interfering with the integrity of the f-actin and microtubular networks altered the overall cell area and found no significant difference in cell area between stretched non-treated and treated cells, independent of the drug we used (Fig. 1E). Measuring cell elongation, we found that cells treated with actin- or MT-targeting drugs were less elongated than nontreated stretched cells (Fig. 1F; *p < 0.05).

In summary, only the mean cell orientation (〈cos 2φ〉) varied between cells that were either investigated under stretched or static conditions (compare Fig. 1D with Fig. S2A, Supporting Information). No differences were observed for the mean cell area and the cell orientation between the different conditions (compare Figs. 1E and 1F with Figs. S2B and S2C, Supporting Information), whereas other studies reported variations in at least one of these parameters [Dartsch and Betz, 1989; Wang et al., 2001]. However, different cell types, stretching devices and protocols were used which might explain the contradictory observations.

In conclusion, our data demonstrate that the stretch-induced cell reorientation is mainly driven by acto-myosin function but MTs can regulate the kinetics of the cell reorientation process.

### The Actin and the Microtubule Network Align Perpendicular to the Stretch Direction

To investigate whether the actin stress fibers and the MT network showed a stretch-induced reorientation response, we stained for actin (phalloidin) and MTs (anti-β-tubulin antibody) under varying conditions (Fig. 2A). The maximum degree of alignment perpendicular to the force axis under nontreated conditions was 〈cos 2φ〉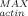 ≍ −0.67> for actin stress fibers and 〈cos 2φ〉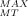 ≍ −0.64> for MTs (Fig. 2B). No stretch-induced perpendicular MT alignment was observed after distortion of actin via cytochalasin D treatment, as reported previously [Goldyn et al., 2009]. Additionally, there was no significant MT reorientation of cells treated with latrunculin B and jasplakinolide (Fig. 2B). However, the orientation of MTs in blebbistatin-treated cells was significantly parallel with respect to the stretch direction (〈cos 2φ〉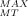 ≍ 0.21) (Fig. 2B; *, *P* < 0.05), similar to the parallel cell body alignment observed at the same conditions (〈cos 2φ〉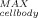 ≍ 0.2) (Figs. 1C and 1D; *, *P* < 0.05). Stabilized MTs in stretched cells reoriented to a degree of 〈cos 2φ〉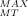 ≍ −0.6>. Cells incubated with taxol or nocodazole had an intact actin stress fiber network which aligned perpendicular to the stretch direction (taxol: 〈cos 2φ〉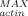 ≍ −0.59; nocodazole: 〈cos 2φ〉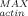 ≍ −0.75). The actin stress fibers in nocodazole-treated cells revealed a slightly but not significantly higher degree of stretch-induced reorientation compared to taxol-treated and nontreated cells (Fig. 2B).

Summing up, the stretch-induced reorientation response of MTs and actin stress fibers coincide largely with the degree of orientation of the cell bodies. These data show that actin is the driving component of cellular rearrangements but MTs can modulate the degree of repolarization of cells under stretching forces.

### The Correlation Between Actin Stress Fiber and Microtubules Orientation Increases When Cells are Subjected to Cyclic Stretching

Stretch-induced perpendicular actin stress fiber and MT orientation seemed to coincide (Figs. 2A and 2B). To analyze the dynamic reorientation of actin stress fibers and MTs in more detail we applied an algorithm to measure the correlation in the local alignment of the two cytoskeleton networks over time. The principle data evaluation is demonstrated in Figure 3A, where orientation lines were plotted into the cellular subareas of images of stained actin and MTs. Overlays of the respective lines were merged into a new image (Fig. 3A). The correlation coefficient between actin stress fibers and MTs was determined (Fig. 3B). Nontreated, nonstretched cells (control conditions) showed only a weak spatial correlation of about 0.24 of actin stress fibers and MT orientation. Upon stretch application [stretch (+)], the correlation coefficient (corr) in nontreated cells increased over time to a maximum of corr ≍ 0.52 which was reached after 3 h of force application (Fig. 3B; *, *P* < 0.05). This increase in correlation between f-actin and MT orientation did not depend on the dynamics and the *de novo* polymerization of MTs, since MT stabilization using taxol showed similar correlations between the two networks. The correlation coefficient of actin stress fiber and MT orientation was calculated in these MT-stabilized cells as corr ≍ 0.28 at nonstretched control conditions and revealed a two-fold increase (corr ≍ 0.59) upon stretching (Fig. 3C; *, *P* < 0.01).

These data suggest an increasing functional association between actin stress fibers and MTs when cells are subjected to stretching forces.

### Stretch-Induced Oriented Migration Requires an Intact Actin and Microtubule Cytoskeleton

The synergy between the actin network and MTs is important for cell migration [Wehrle-Haller and Imhof, 2003; Etienne-Manneville, 2004; Li et al., 2005]. Additionally, it has been shown that physical forces (e.g. shear flow) and mechanical properties of a surface (e.g. stiffness) can influence cell migration [Pelham and Wang, 1997; Shiu et al., 2004; Discher et al., 2005; Li et al., 2005]. We have previously shown that MTs are essential for cell motility under stretching forces [Goldyn et al., 2009]. We have extended these studies and now tested the reactions of cells upon interference with the actin network by drugs. To quantify cell motility, we calculated a migration index whereby a value of 1 indicates random migration, a value smaller than 1 indicates cell migration parallel and a value greater than 1 perpendicular to the stretch axis. Stretched nontreated cells migrated preferentially perpendicular to the stretch direction with a migration index of 1.7 (Fig. 4A, *, *P* < 0.01). The overall distance that nontreated cells migrated within 8 h was not significantly different under nonstretching (≍ 170 μm/8 h) and stretching conditions (≍ 200 μm/8 h) (Fig. 4B; ‡, *P* > 0.05). The directionality of cell migration for nontreated cells did not significantly differ under nonstretched and stretching conditions (black bars in Fig. 4C). All types of actin-interfering drugs prevented directed migration perpendicular to the stretch direction (Fig. 4A compared to Fig. S3A, Supporting Information). Stretching did not affect cell motility under actin-treated and MT-treated conditions (Fig. 4B and Fig. S3B, Supporting Information). However, stretching increased the migration of cells treated with blebbistatin. These cells with inhibited myosin II became faster upon stretching and migrated similar distances as nontreated cells (distance_blebb_ ≍ 180 μm/8 h; ‡, *P* > 0.05) (Fig. 4B). Cyclic stretching did not influence the directionality of migration in cells treated with actin-interfering drugs (Fig. 4C and Fig. S3C, Supporting Information). Examples of cell migration tracks under stretching and nonstretching conditions are given in Figure 4D and Figure S3D, Supporting Information.

These data suggest that both types of cytoskeleton, actin, and MTs, need to be intact for coordinated, oriented cell migration under stretching forces.

## Discussion

In this study we showed that actin drives the process of cellular reorientation upon application of directional forces and that microtubules (MTs) can modulate the kinetics of the responses. All actin perturbing reagents used were potent inhibitors of repolarization of cells under stretching conditions. We previously revealed that MTs are not essential for the extent of cellular reorganization [Goldyn et al., 2009]. Now we demonstrate that the disruption of MTs leads to an accelerated cellular reorientation, whereas stabilization of MTs slows this process down. One explanation could be that RhoA activation upon cyclic stretching sensitizes cells to mechanical stimuli. The effect of RhoA activation is increased by disrupting MTs with nocodazole. This is known to enhance increased intracellular tension and actin stress fiber formation [Bershadsky et al., 1996; Enomoto, 1996; Goldyn et al., 2009]. MT stabilization using taxol, however, does not further increase the stretch-induced RhoA activation [Goldyn et al., 2009]. The altered RhoA activation modifies the acto-myosin controlled cell contractility which is thought to be crucial for force-transduction and mechano-response upon stretching [De et al., 2007]. This idea is supported by our data showing that the inhibition of the acto-myosin machinery using blebbistatin prevents polarized reorientation induced by stretching forces.

An alternative explanation of the different kinetics observed upon MT disruption or stabilization could be the possible effect of MTs sterically hindering the actin reorganization. It is known that MTs are highly dynamic structures, undergoing growth and catastrophe events allowing the rapid reorganization of the entire network [Mikhailov and Gundersen, 1998]. However, MTs are also stiff elements and thus build a rigid frame that can resist external and internal forces [Stamenovic et al., 2002]. Danowski has put forward the idea that MTs may exert a pushing force, which partly counterbalances actin-generated contractile forces [Danowski, 1989]. In a simplified model stabilized microtubules might just be a barrier for the reorganization of actin; a disruption of MTs on the other hand gives way to an efficient, fast reorganization. This idea of MTs influencing the kinetics of the cell reorientation process was also supported by the observation that MTs may homogenize the strain distribution in *in vitro* actin networks and thus work as global stabilizing elements [Gardel et al., 2008].

A third possibility of how MTs influence the kinetics of cell repolarization may rely on the tight association of MTs with the f-actin network through cross-linking proteins such as spectraplakins, formins and others [Goode et al., 2000; Ishizaki et al., 2001; Kodama et al., 2003]. The fact that (intact and stabilized) MTs align with the actin fibers perpendicular to the direction of stretch might be explained by the cross-linking of MTs with actin fibers. The acto-myosin system seems to lead the stretch-induced MTs and cell reorientation. Subsequently, the acto-myosin network would need to drag MTs and this would delay the stretch-induced cell reorganization.

A surprising and intriguing observation in our study was that we could restore cell migration speed of myosin II-inhibited cells by the application of stretching forces. It is possible that signaling mechanisms that drive cell migration were stimulated by the outside application of forces. We speculate that formins such as mDia1 might be involved in this phenomenon. This protein was shown to be involved in focal adhesion and actin polymerization events when forces were applied from the outside of cells [Riveline et al., 2001]. In analogy to this experiment we assume that mDia might be able to compensate for the loss of internal tension mediated by downstream events of RhoA (i.e. myosin activity). If mDia - as the current models suggest - acts as a leaky cap at the cell edges that may respond to forces promoting localized actin polymerization via RhoA [Watanabe and Higashida, 2004], it would be an ideal candidate driving such force-mediated motility.

Our observation that blebbistatin-treated cells tend to orient slightly parallel to the stretch direction would be in line with the hypothesis of mDia acting as a leaky cap. If actin polymerization predominantly occurs at sites of forces then actin polymerization would preferentially occur at cellular ends pointing in the direction of the applied forces. As a consequence, cells would elongate slightly along this force direction. However, further experiments will be needed to confirm this hypothesis.

## Conclusion

In an advancement to previous reports, we demonstrated that the kinetics of the stretch-induced cellular reorientation are influenced by MTs, although they show only minor reorganization in absence of the actin network. The increased stretch-induced local co-alignment of actin stress fibers and MTs, even after taxol treatment, indicates that actin is the driving filament structure in the cellular reorientation process; the speed of this process, however, depends on MTs. Thus the MT system might act as a global stabilizer in cells if they are subjected to uniaxial cyclic stretching. Furthermore, our study revealed that the reduced cell migration due to myosin II-inhibition by blebbistatin could be rescued by stretch application. We speculate that mDia1 is involved in this phenomenon by compensating for the loss of cell internal tension induced by blebbistatin treatment.
