# Supplementary material for: The Kinetics of Force-Induced Cell Reorganization Depend on Microtubules and Actin
Source: Cytoskeleton (Hoboken). 2010 Feb 26;67(4):241–50. doi: 10.1002/cm.20439 (PMC3638371; doi:10.1002/cm.20439)
Supplement: Supplementary file 1 [file cm0067-0241-SD1.doc]

**Supplemental Data**

**Table SI:**

List of pharmacological cytoskeleton inhibitors used in the presented study. The target of each used drug and its specific function is listed together with several references for more details. For an overview of chemical inhibitors of the cytoskeleton see Peterson *et al.* (Peterson and Mitchison 2002).

| Treatment | Direct or indirect target | Function | References |
| --- | --- | --- | --- |
| Latrunculin B (Lat B) | Actin | forms 1:1 complexes with actin monomers and blocks actin polymerization | (Wakatsuki et al. 2001) |
| Cytochalasin D (Cyto D) | Actin | binds to the barbed ends of actin polymers and inhibits actin polymerization | (Wakatsuki et al. 2001) |
| Jasplakinolide (Jaspla) | Actin | enhances actin polymerization/actin nucleation | (Bubb et al. 2000) |
| Blebbistatin (Blebb) | Actin (via myosin II) | inhibits myosin II activity and subsequently actin fibre cross-linking | (Allingham et al. 2005; Kovacs et al. 2004) |
| Nocodazole (Noco) | Microtubules | disrupts the microtubule network by depolymerizing them | (Bershadsky et al. 1996; Mikhailov and Gundersen 1998) |
| Taxol (Tax) | Microtubules | abolished microtubule dynamics by stabilizing microtubules (hyperpolymerization) | (Green and Goldman 1983; Mikhailov and Gundersen 1998) |

**References for Table S1:**

Allingham JS, Smith R, Rayment I. 2005. The structural basis of blebbistatin inhibition and specificity for myosin II. Nat Struct Mol Biol 12(4):378-9.

Bershadsky A, Chausovsky A, Becker E, Lyubimova A, Geiger B. 1996. Involvement of microtubules in the control of adhesion-dependent signal transduction. Curr Biol 6(10):1279-89.

Bubb MR, Spector I, Beyer BB, Fosen KM. 2000. Effects of jasplakinolide on the kinetics of actin polymerization. An explanation for certain in vivo observations. J Biol Chem 275(7):5163-70.

Green KJ, Goldman RD. 1983. The effects of taxol on cytoskeletal components in cultured fibroblasts and epithelial cells. Cell Motil 3(4):283-305.

Kovacs M, Toth J, Hetenyi C, Malnasi-Csizmadia A, Sellers JR. 2004. Mechanism of blebbistatin inhibition of myosin II. J Biol Chem 279(34):35557-63.

Mikhailov A, Gundersen GG. 1998. Relationship between microtubule dynamics and lamellipodium formation revealed by direct imaging of microtubules in cells treated with nocodazole or taxol. Cell Motil Cytoskeleton 41(4):325-40.

Peterson JR, Mitchison TJ. 2002. Small molecules, big impact: a history of chemical inhibitors and the cytoskeleton. Chem Biol 9(12):1275-85.

Wakatsuki T, Schwab B, Thompson NC, Elson EL. 2001. Effects of cytochalasin D and latrunculin B on mechanical properties of cells. J Cell Sci 114(Pt 5):1025-36.

**Figure S1**


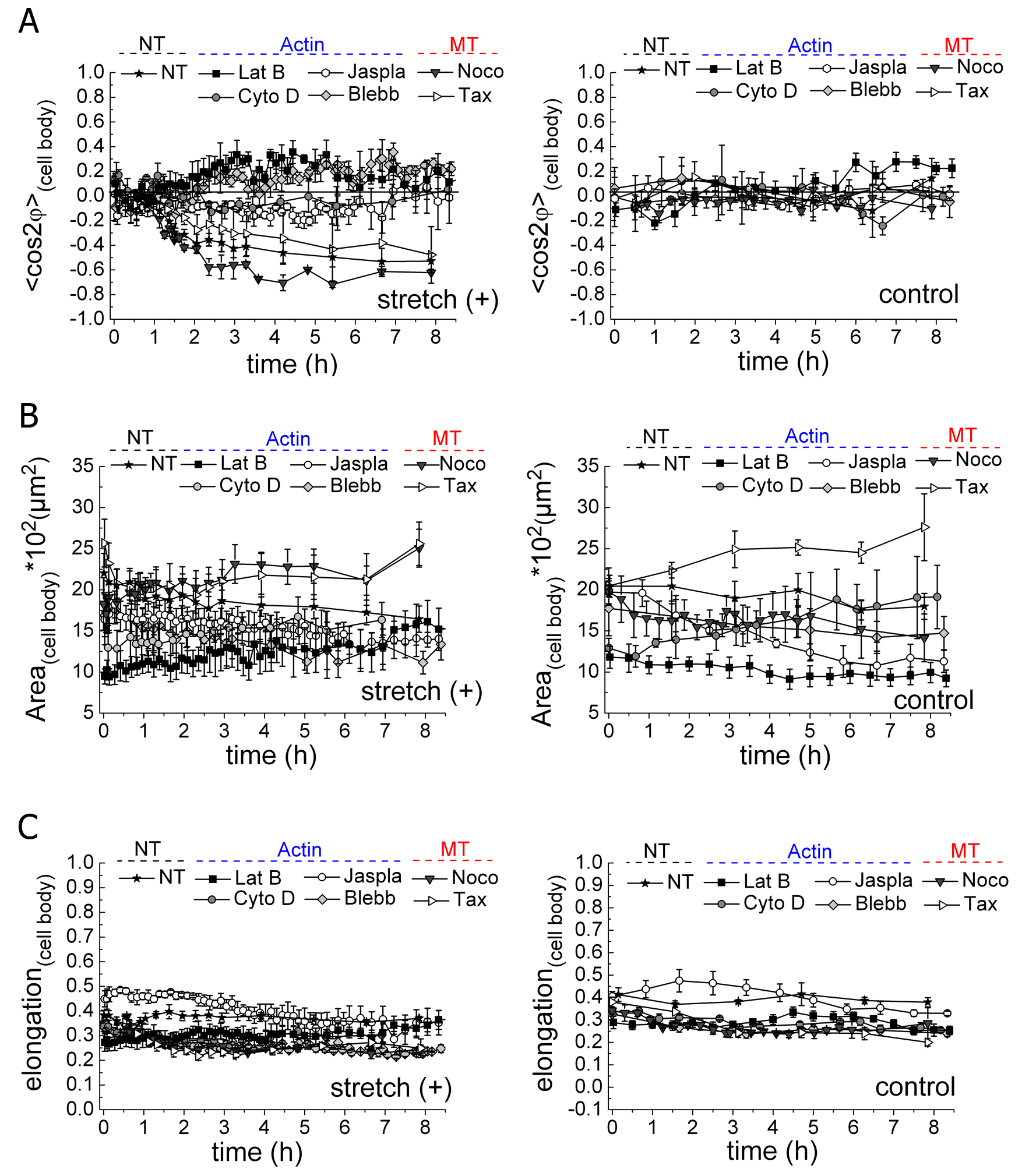


**Figure S1**

**(A)** Time-response of the mean reorientation of cells upon uniaxial cyclic stretching of 8 % at 1 Hz (stretch (+)) and non-stretched control conditions. The mean value of <cos2φ> = 1 indicates that the cells are in average perfectly parallel orientated, the minimum of -1 indicates a perfectly perpendicular alignment of an average of cells with respect to the stretch direction. A value of <cos2φ = 0> corresponds to a random orientation of cells.

**(B)** Time-response of the average cell area under cyclic stretch (stretch (+)) and non-stretched control conditions. The cell area was determined by outlining of the cell shape.

**(C)** Time-response of the mean cell elongation under cyclic stretch (stretch (+)) and non-stretched control conditions. The cell elongation was given by the ratio of the long and the short cell axis of the cell.

NT = non-treated cells; Actin = cells with a disturbed actin network (Lat B = latrunculin B; Cyto D = cytochalasin D; Jaspla = jasplakinolilde; Blebb = blebbistatin) MT = cells with a modified microtubule network (Noco = nocodazole; Tax = taxol).

**Figure S2:**


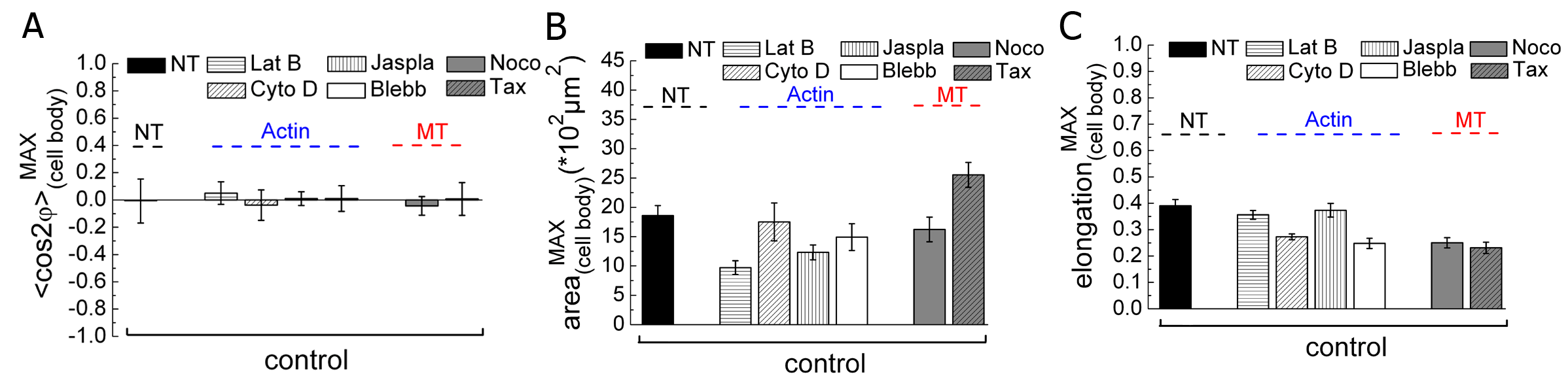


**Figure S2:**

**(A)** Quantification of the maximum mean cell reorientation (<cos2φ>) under the conditions indicated. Control indicates non-stretched experimental conditions.

**(B)** Quantification of the maximum mean cell area (area) under the non-stretched (control) conditions.

**(C)** Quantification of the maximum mean cell elongation (elongation) under the non-stretched (control) conditions. A value of 0 corresponds to a spherical cell area; a value close to 1 represents an extremely elongated cell.

NT = non-treated cells; Actin = cells with a disturbed actin network (Lat B = latrunculin B; Cyto D = cytochalasin D; Jaspla = jasplakinolide; Blebb = blebbistatin); MT = cells with a modified microtubule network (Noco = nocodazole; Tax = taxol).

**Figure S3**


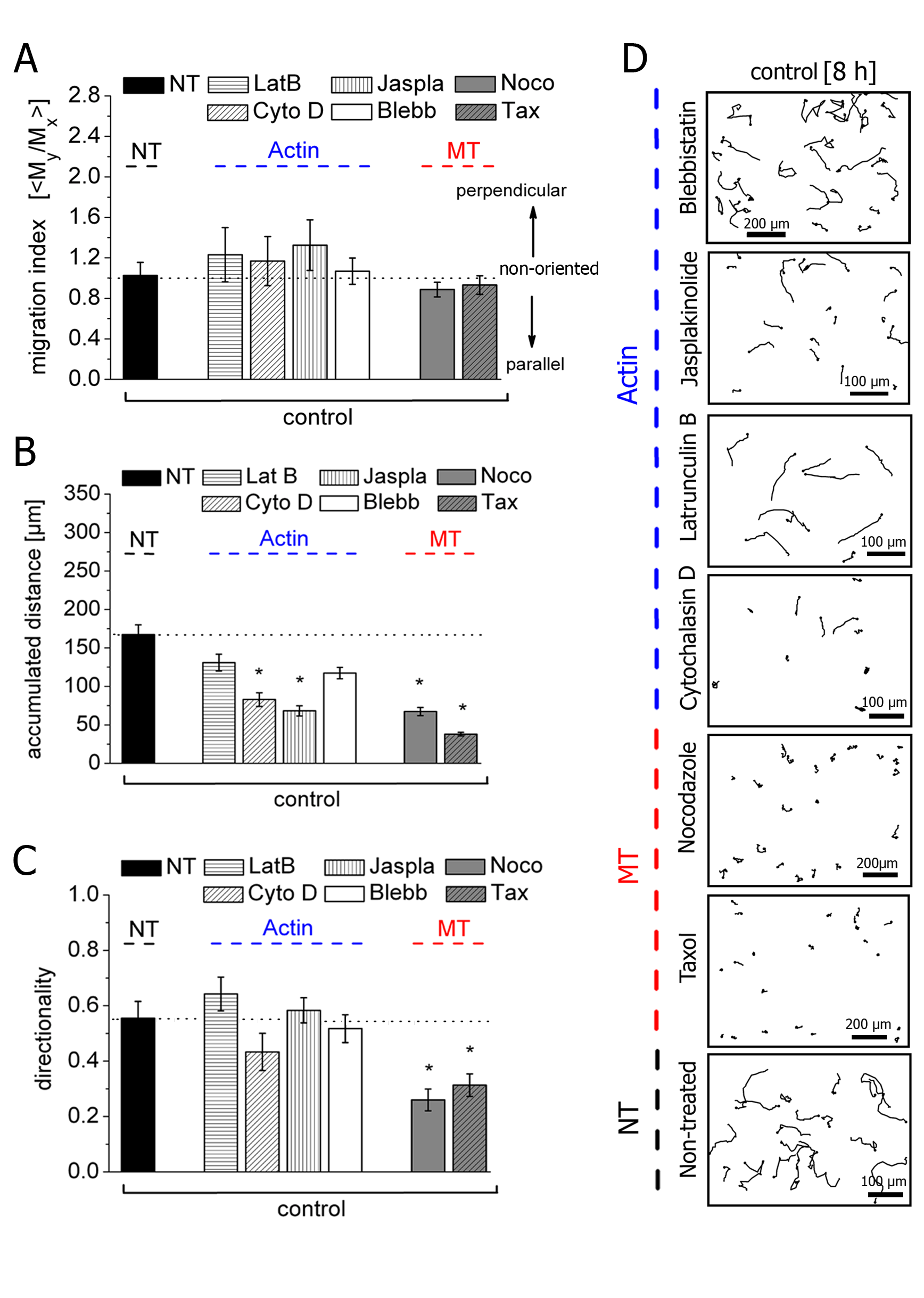


**Figure S3**

**(A)** Quantification of oriented cell migration was performed over 8 hours under non-stretched control conditions. A migration index M_y_/M_x_ = 1 means that the cells showed no preferred migration direction (black dotted line in the graph), for M_y_/M_x_ < 1 cells migrate preferentially parallel to the stretch direction, and for M_y_/M_x_ > 1 cells migrate preferentially perpendicular to the stretch direction.

**(B)** Quantification of accumulated distance of cell migration under non-stretched (control) conditions. The asterix indicates the experimental conditions where the distance of cell migration was significantly different compared to non-treated conditions (*, p<0.05).

**(C)** Quantification of the directionality of migration of non-stretched control cells. A value of 1 for the directionality would mean that cells migrate in a straight line. Cells treated with nocodazole or taxol, respectively, reveal a significant difference in the directionality of their migration compared to non-treated cells (*, p<0.05).

**(D)** Tracks of migrating cells were recorded for 8 hours under non-stretched conditions. Migration was abolished after treatment of cells with taxol or nocodazole to stabilize or disrupt microtubules, respectively (MT, red dotted line). The direction of stretch is indicated by the black double-headed arrow.
